# Tree Diversity Limits the Impact of an Invasive Forest Pest

**DOI:** 10.1371/journal.pone.0136469

**Published:** 2015-09-11

**Authors:** Virginie Guyot, Bastien Castagneyrol, Aude Vialatte, Marc Deconchat, Federico Selvi, Filippo Bussotti, Hervé Jactel

**Affiliations:** 1 INRA, DYNAFOR, UMR 1201, 31326 Castanet Tolosan, France; 2 Université de Toulouse, INPT-ENSAT, DYNAFOR, UMR 1201, 31326 Castanet Tolosan, France; 3 Université de Bordeaux, BIOGECO, UMR 1202, 33405 Talence, France; 4 INRA, BIOGECO, UMR 1202, 33610 Cestas, France; 5 Università di Firenze, DISPAA, Laboratori di Botanica, 50144 Florence, Italy; University of Oxford, UNITED KINGDOM

## Abstract

The impact of invasive herbivore species may be lower in more diverse plant communities due to mechanisms of associational resistance. According to the “resource concentration hypothesis” the amount and accessibility of host plants is reduced in diverse plant communities, thus limiting the exploitation of resources by consumers. In addition, the “natural enemy hypothesis” suggests that richer plant assemblages provide natural enemies with more complementary resources and habitats, thus promoting top down regulation of herbivores. We tested these two hypotheses by comparing crown damage by the invasive Asian chestnut gall wasp (*Dryocosmus kuriphilus*) on chestnut trees (*Castanea sativa*) in pure and mixed stands in Italy. We estimated the defoliation on 70 chestnut trees in 15 mature stands sampled in the same region along a gradient of tree species richness ranging from one species (chestnut monocultures) to four species (mixtures of chestnut and three broadleaved species). Chestnut defoliation was significantly lower in stands with higher tree diversity. Damage on individual chestnut trees decreased with increasing height of neighboring, heterospecific trees. These results suggest that conservation biological control method based on tree species mixtures might help to reduce the impact of the Asian chestnut gall.

## Introduction

Biological invasions have been responsible for more than 74% of known species extinctions [1,2], being one of the major causes of biodiversity loss. For example, two temperate forest tree species came close to extinction in the 20^th^ century due to exotic pathogens: the American chestnut (*Castanea dentata* (Marsh.) Borkh) due to chestnut blight introduced from Asia, and the European elms following the introduction of elm disease from North America. Currently, the emerald ash borer (*Agrilus planipennis* Fairmaire), which was introduced from Asia, is threatening the survival of the *Fraxinus* genus in the US [3]. The paradox is that biodiversity is itself considered a main driver of resistance to invasion. Since the seminal work of Elton [4], it is commonly assumed that ecosystem invasibility, i.e. susceptibility to invasion by non-resident species [5], decreases with increasing species diversity [6–8]. For example, based on 120 case studies, Cardinale et al. [9] showed that generally, resistance to plant invasion significantly increases with species richness in plant communities. However, most studies of diversity-invasibility relationships were undertaken in grasslands and dealt with plant invasions, and little is known about invasibility by insect herbivores [10], even less in forest ecosystems [11].

An increasing body of evidence supports the “associational resistance hypothesis” [12], which states that more diverse plant associations are less prone to insect damage [13], including in forests [14–16]. Interestingly, the main mechanisms that have been suggested to explain resistance to invasion mirror those underlying associational resistance.

First, according to the “resource availability hypothesis”, ecosystems with higher amount of resources would be more prone to invasion by new species of consumers [17]. Likewise the density of host plants (i.e. the number of individuals) or their relative frequency among neighboring non-host plants are key determinants of herbivory [18,19] in particular by specialist herbivores [16]. For such herbivores, the “resource concentration hypothesis” [20] posits that species-rich plant communities make host plants harder to locate and to reach by their herbivores by diluting them among non-host plants [16,21,22]. Because the higher the number of non-host plants mixed with host plants, the more diluted the resource, associational resistance would therefore be expected to increase with increasing plant diversity.

The second mechanism that may be responsible for the invasion success of exotic plants is known as “the enemy release hypothesis”. It states that once introduced in a new area, exotic plants may escape the top-down control by their specialist natural enemies (primary consumers) [23–25]. Within the associational resistance framework, the “natural enemies hypothesis” predicts a greater top-down control of herbivores in species-rich plant communities by secondary consumers. It may counter the effects of enemy release, suggesting that richer plant assemblages offer a greater array of complementary food and habitat resources that benefit predators and parasitoids [20,26]. Because abundance and species richness of natural enemies usually increase with plant diversity [27,28], richer plant communities may be less invasible by exotic herbivores due to better biological control [29].

There are thus strong reasons to assume that richer plant communities should be less invasible by exotic plants or insect herbivores, not only because of the reduction of resource availability, but also because of more efficient top-down control. Conversely, the “environmental heterogeneity hypothesis” suggests that invasibility by alien plants increases with ecosystem diversity due to higher habitat or resources diversity allowing better coexistence between native and exotic species, if the latter are able to exploit local resources [5]. In the same way “the associational susceptibility hypothesis” [30] has been proposed to account for higher herbivore damage in more diverse plant communities as a result of the possibility for some insects, mainly polyphagous, to shift from one host plant onto the other and hence benefit from a large array of feeding resources [31].

Mechanisms underlying the relationship between plant diversity and resistance to native herbivores may then also impart resistance to non-native herbivores [32]. However, because introduced species did not evolve in interaction with local host plants and natural enemies, they may not respond in the same way to local biotic interactions driven by producer diversity [33]. Addressing this question is important to predict the risk of the establishment and development of alien insects in relation with plant diversity in the invaded area.

In the present study, we looked for associational resistance to the Asian chestnut gall wasp, *Dryocosmus kuriphilus* Yasumatsu (Hymenoptera Cynipidae). We monitored the degree of damage caused by this invasive pest to the European chestnut tree (*Castanea sativa* Mill.) in pure and mixed stands. *Dryocosmus kuriphilus* is a univoltine monophagous herbivore that is a native of China and feeds on species of the *Castanea* genus. It was introduced in Japan in the 1940s [34], and subsequently in South Korea and the United States in the 1970s. Its first occurrence in Europe was reported in 2002, in North West Italy [35]. Since then it has spread to neighboring countries (France, Croatia, Slovenia, Switzerland, Austria, Czech Republic, Germany, Hungary and the Netherlands [36]). After adult emergence in early summer, female wasps lay eggs in buds. First instar larvae overwinter within buds. The following spring, when buds start to develop, growth of second instar larvae induces galls on buds, leaves and shoots [34,37]. Gall forming causes the reduction of leaf area, resulting in a decline of photosynthetic capacity [38]. Severe gall infestations can then affect tree growth and even cause tree mortality [34,37]. Nut yield can be reduced by up to 80% [39]. It has been also suggested that attacks by *D*. *kuriphilus* may enhance dieback caused by ink disease (*Phytophthora cinnamomi* Rands) or chestnut blight fungus (*Cryphonectria parasitica* (Murr.) Barr) [35,40]. Classical biological control of populations of the Asian chestnut gall wasp using *Torymus sinensis* Kamijo, a parasitoid species from the native range of *D*. *kuriphilus*, has been tried in Italy with equivocal success [35]. But recently, observations made both in North America [41] and Italy [42–46] indicate that native parasitoids, in particular those of oak-galling cynipids, can shift onto the invasive chestnut gall wasp [47].

Here, we focused on the recent expansion of *D*. *kuriphilus* in Italian forests. By measuring chestnut tree infestation along a gradient of tree diversity from pure stands of *C*. *sativa* to mixtures in natural mature forests [48], we tested the following predictions:

1. Gall damage by *D*. *kuriphilus* decreases with the diversity of tree species associated with *C*. *sativa*;
2. the level of gall wasp damage is lower where chestnut trees are more diluted among non-conspecific neighbors (“the resource concentration hypothesis”);
3. chestnut infestation by *D*. *kuriphilus* is reduced in the presence of other *Fagaceae* species hosting cynipid galls and associated generalist parasitoids (“the natural enemies hypothesis”). Recently several studies revealed that reduced plant apparency was an overlooked driver of associational resistance [16,49] as focal trees hidden amongst heterospecific trees are less likely to be found by insect herbivores. We therefore propose a fourth prediction, i.e. that
4. the presence of taller non-conspecific neighboring trees reduces the amount of gall wasp damage on smaller chestnut trees (“the plant apparency hypothesis”).

## Materials and Methods

### Site description

The present study emerged from a global survey of tree diversity effect on insect herbivory in European mature forests (FunDivEUROPE project, www.fundiveurope.eu). It was carried out in southern Tuscany (province of Siena, Italy) where the Asian chestnut gall wasp was established since 2008 [38], offering the opportunity to test the associational resistance hypothesis with an invasive forest insect.

Fifteen 30 m × 30 m comparative plots were selected in deciduous mature forests (Alto-Merse N43°10'11.58'' E11°12'7.98'' and Belagaio N43°4'46.92'' E11°13'30.6'' forests) along a gradient of tree species diversity (see Baeten et al. [48] for details). Within the FunDivEUROPE project framework, site managers had beforehand obtained permission by private or communal owners to conduct the study on each plot. No endangered or protected species were collected or destructed during the field campaign. All plots contained *C*. *sativa* trees, alone (i.e. monocultures) or associated with one to four of the following broadleaved species: *Ostrya carpinifolia* Scop., *Quercus cerris* L., *Q*. *ilex* L. and/or *Q*. *petraea* (Matt.) Liebl.. Chestnut trees in our study area belong to the same local provenance. Current chestnut forest stands originate in the reverse conversion to coppice of old chestnut orchards (following chestnut blight attacks in the last century) that were originally obtained by grafting local wild chestnut trees. The location within the plot, tree species identity, stem diameter at breast height (DBH), crown diameter and the height of all the trees were measured in each plot. Table 1 summarizes the characteristics of the sampled plots and trees along the gradient of tree species richness.

**Table 1:** Characteristics of chestnut plots and trees sampled along the gradient of tree species richness.

Tree species richness(no. of plots)	Number of sampled *Castanea sativa* trees		Mean characteristics of sampled *Castanea sativa* trees			Number of plots with another tree species			
	Crown assessment	Leaf assessment	Height (m)	Basal area (m^2^)	Crown area (m^2^)	*Quercus cerris*	*Quercus ilex*	*Quercus petraea*	*Ostrya carpinifolia*
1 (2)	19	12	15.21 ±3.35	0.03 ±0.03	14.05 ± 8.74	**0**	**0**	**0**	**0**
2 (3)	14	9	14.51 ±3.14	0.03 ±0.03	16.57 ± 10.06	**1**	**1**	**0**	**1**
3 (5)	18	15	14.57 ±2.89	0.02 ±0.02	12.72 ± 7.72	**3**	**3**	**3**	**1**
4 (5)	19	15	15.92 ±3.51	0.04 ±0.05	22.13 ± 17.25	**4**	**5**	**3**	**3**

### Assessment of damage caused by *D*. *kuriphilus*

From June 12 to June 27, 2012, the crowns of a total of 70 chestnut trees (hereafter referred to as focal trees) were assessed. Before the field campaign, 51 focal chestnut trees were randomly preselected from plot maps. Three focal chestnut trees were chosen in each mixed plot among the six trees with the largest DBH and six individual chestnut trees among the 12 largest ones in pure stands.

Our protocol for crown condition survey was derived from the ICP Forests manual [50], adapted to be better account for total insect damage. One of the main differences was that insect damage was assessed on the whole crown, instead of the “assessable crown” only. Damage was thus assessed separately in the parts of the crown exposed to sunlight and in the shade, as foliar loss may be also due to competition for light or natural pruning in the shaded part, given that *C*. *sativa* is heliophilous. We considered damage as leaf area reduction due to *D*. *kuriphilus* galls, hereafter termed as defoliation. To assess defoliation, a comparison was made between the focal tree and a “reference tree”, i.e. a healthy tree with full foliage, according to the ICP Forests manual. We recorded the respective proportion of the crown exposed to sunlight (*P*_*CL*_), the proportion of dead branches in the parts of the crown exposed to sunlight (*P*_*DBL*_), and those in the shade (*P*_*DBS*_), and the proportion of defoliation in the living crown (i.e. the crown excluding the dead branches) in the part exposed to sunlight (*P*_*DL*_), and in the shade (*P*_*DS*_) respectively. The number of branches was counted in order to better estimate the proportion of dead branches in each part of the crown. The following percentage classes were used for all proportion variables: 0%, 0.5–1%, 1.5–12%, 12.5–25%, 25.5–50%, 50.5–75% and > 75%. The assessment was done from at least two sides of the crown to account for all damage. Where a different score was attributed to a focal tree from different sides, the mean of damage class median was used.

The total proportion of dead branches in the part of the crown exposed to sunlight was then calculated as:

$$T_{DBL}=P_{CL}\times P_{DBL}$$ Eq 1

The total percent of defoliation was estimated as:

$$T_{D}=P_{ACL}\times P_{DL}+\left(1-P_{ACL}\right)\times P_{DS}$$ Eq 2

where *P*_*ACL*_ represents the proportion of the living crown exposed to sunlight:

$$P_{ACL}=\frac{P_{CL}\left(1-P_{DBL}\right)}{P_{CL}\left(1-P_{DBL}\right)+\left(1-P_{CL}\right)\left(1-P_{DBS}\right)}$$ Eq 3

The total proportion of damaged crown was then calculated as:

$$T_{DC}=P_{DL}\times P_{CL}\left(1-P_{DBL}\right)+P_{DS}\left(1-P_{CL}\right)\left(1-P_{DBS}\right)+T_{DBL}$$ Eq 4

Fig A in S1 File gives a schematic representation of tree crown, illustrating the different variables used to quantify Asian gall wasp damage (see Supporting Information).

In addition, herbivory by *D*. *kuriphilus* was assessed on leaves, to confirm that crown damage was due to cynipid galls. Two branches per sampled chestnut tree were cut by tree climbers, one at the top and the other in the middle of the crown, in the part exposed to sunlight. Nineteen trees that had been randomly preselected could not be climbed for safety reasons. The closest climbable tree was then chosen and crown damage was re-assessed on these additional trees. Thirty leaves were collected at random on each cut branch and frozen at -18°C until assessment. Attacks by *D*. *kuriphilus* were estimated using the percentage of leaves with at least one gall. Damage was then aggregated at the tree level by calculating the percentage of leaves impacted by at least one gall of *D*. *kuriphilus*.

### Estimation of the abundance of native galls on oak trees present in mixed stands

As a Cynipidae, *D*. *kuriphilus* may share natural enemies with other native cynipid gall makers [42,46]. Thus, in addition to estimating the abundance of galls made by *D*. *kuriphilus* on chestnut trees, leaf collection was used to estimate the abundance of cynipid galls on *Quercus spp*. in mixed plots. We used the same method as for chestnuts for tree selection and leaf collection. We counted the number of leaves with at least one gall made by Cynipidae gall makers on 24 *Q*. *cerris* trees (1440 leaves), 27 *Q*. *ilex* (1640 leaves) and 17 *Q*. *petraea* (1060 leaves). Then we aggregated damage at the tree level by calculating the percentage of leaves with at least one gall. As we did not rear parasitoids, we were not able to directly estimate the percentage of parasitized galls. We therefore used the percentage of oak leaves with a cynipid gall as a proxy of parasitoids potential abundance in tree mixtures.

### Tree diversity and apparency variables

Explanatory variables were defined at both the plot and the neighborhood levels.

At the plot level, we used the tree species richness, Shannon’s index of tree diversity, and the proportion of *C*. *sativa*, the latter two being based on relative stem basal area. Taxonomic diversity was also used to quantify the taxonomic distance between different tree species in the plot. The index was calculated as the average length of the path (i.e. average distance) connecting two individual tree species, traced through a Linnaean classification of the full set of species in the tree sample [51], using the *taxondive* function in the *vegan* package (version 2.0–10) in *R* [52].

For the neighborhood level, we considered neighbors all trees whose crown was within a 3 m radius of that of the focal tree. Coppice clumps were considered a single tree. The list of 506 neighboring trees was extracted with ArcMap and ArcToolbox software, ArcGis for Desktop version 10 ArcInfo advanced [53], using tree position and crown area projection from plot maps. Based on this list, we calculated the same explanatory variables as at the plot level. In addition, to account for structural heterogeneity due to differences in tree height among focal and neighboring trees, we calculated an index of chestnut tree apparency (*ΔH*) based on Castagneyrol et al. [49]:

$$\Delta H=\left(\frac{1}{N}\right)\times \sum_{i}\left(H_{focal}-H_{neighbor\;i}\right)$$ Eq 5

where *H*_*focal*_ and *H*_*neighbor i*_ are the height of the focal tree and of the i^th^ neighboring tree out of *N*. Focal trees that were on average taller and shorter than their neighbors had positive and negative tree apparency *ΔH*, respectively.

Overall, 70 focal chestnut trees were included in analyses at the plot level. Only 31 of these trees were used at the neighborhood level because the other sampled chestnut trees were located at the margin of sampled plots and no information was available on their neighbors growing outside the plot.

### Statistical analyses

The total percent of defoliation (*T*_*D*_, referred as total defoliation) was strongly correlated to the total proportion of damaged crown (*T*_*DC*_, referred as total damaged crown) (correlation *T*_*D*_-*T*_*DC*_: *n* = 70, Pearson’s *r* = 0.81, *P* < 0.001). Because branch mortality may be due to other factors than attacks by *D*. *kuriphilus*, total defoliation was preferred to total damaged crown as a response variable. Total defoliation was positively and significantly correlated with the mean percentage of leaves with at least one gall (*n* = 49, Pearson’s *r* = 0.47, *P* < 0.001). However the later damage measurement was only assessed on two branches per tree and thus considered less accurate than total defoliation_,_ which was therefore used as herbivory response variable in all analyses.

Before performing any formal analyses, data structure was explored following recommendations by Zuur et al. [54]. Cleveland dot plots of total defoliation identified potential outliers, which were further checked by simulating 1,000 random samples from normal distribution with sample size, mean and standard deviation taken from raw data [54]. Data points falling outside the 95% confidence interval derived from these simulations were considered as true outliers, and four trees were then discarded from analyses at the plot level, and one tree at the neighborhood level. However analyses were redone after reincorporating outliers to check for consistency in pattern of responses.

Analyses at the plot and the neighborhood levels were carried out separately, but using the same modelling approach. Trees were used as statistical units. We used linear mixed effects models (*lmer* function in the *lme4* package version 1.1–7 in *R* [55]), with the plot as random factor to account for pseudo-replication of trees within plots. A *log* transformation was applied to total defoliation to satisfy the assumptions of statistical tests.

The five explanatory variables at the plot level (i.e. tree species richness, Shannon’s diversity index, the proportion of *C*. *sativa*, the index of taxonomic diversity, and the percentage of oak leaves with galls of Cynipidae) were strongly correlated (all pairwise correlations with Pearson’s *r* > 0.42), preventing the use of multiple regressions [56,57]. Univariate models were then preferred and compared in an information theory approach. We first built a set of five univariate models, *plus* the null model (i.e. intercept only). The set of best fitting models was selected based on Akaike’s information criterion, corrected for small sample sizes (AICc [58]) using the *selMod* function in the *pgirmess* package (version 1.5.9) in *R* [59]. Among the best fitting models, the minimum adequate model (MAM), i.e. most parsimonious model, was that with the lowest number of estimable parameters (*K*) within 2 AICc units of the model with the lowest AICc. Differences in AICc scores (*Δ*_*i*_) > 2 are usually interpreted as indicating strong support for the MAM compared to poorer models [58]. Estimates of model parameters are reported for the MAM.

The same approach was used at the neighborhood level including the same five explanatory variables used at plot level. Because tree apparency was not correlated with tree richness or taxonomic diversity at this level (respectively Pearson’s *r* = -0.30, *P* = 0.09 and Pearson’s *r* = -0.06, *P* = 0.72), it was possible to add multivariate models to the analysis. We considered the null model, five univariate models with explanatory variables at plot level (see above), univariate models with explanatory variables at the neighborhood level (i.e. tree species richness, Shannon’s diversity index, the proportion of *C*. *sativa*, taxonomic diversity and tree apparency), and four multivariate models including tree apparency *ΔH*, tree species richness or taxonomic diversity, and their interaction. Then we compared these 15 models using the same method as at the plot level. All statistical analyses were performed with *R* free software [60].

All data used for statistical analyses are reported in Table A in S1 File and *R* syntax of each model is detailed in Table B in S1 File (see Supporting Information).

## Results

Observations showed that 100% of the chestnut trees that we sampled were attacked by *D*. *kuriphilus*. Total defoliation caused by this invasive pest was on average 12.7 ± 8.1%, ranging from 0.8% to 31.1%.

**Fig 1:**
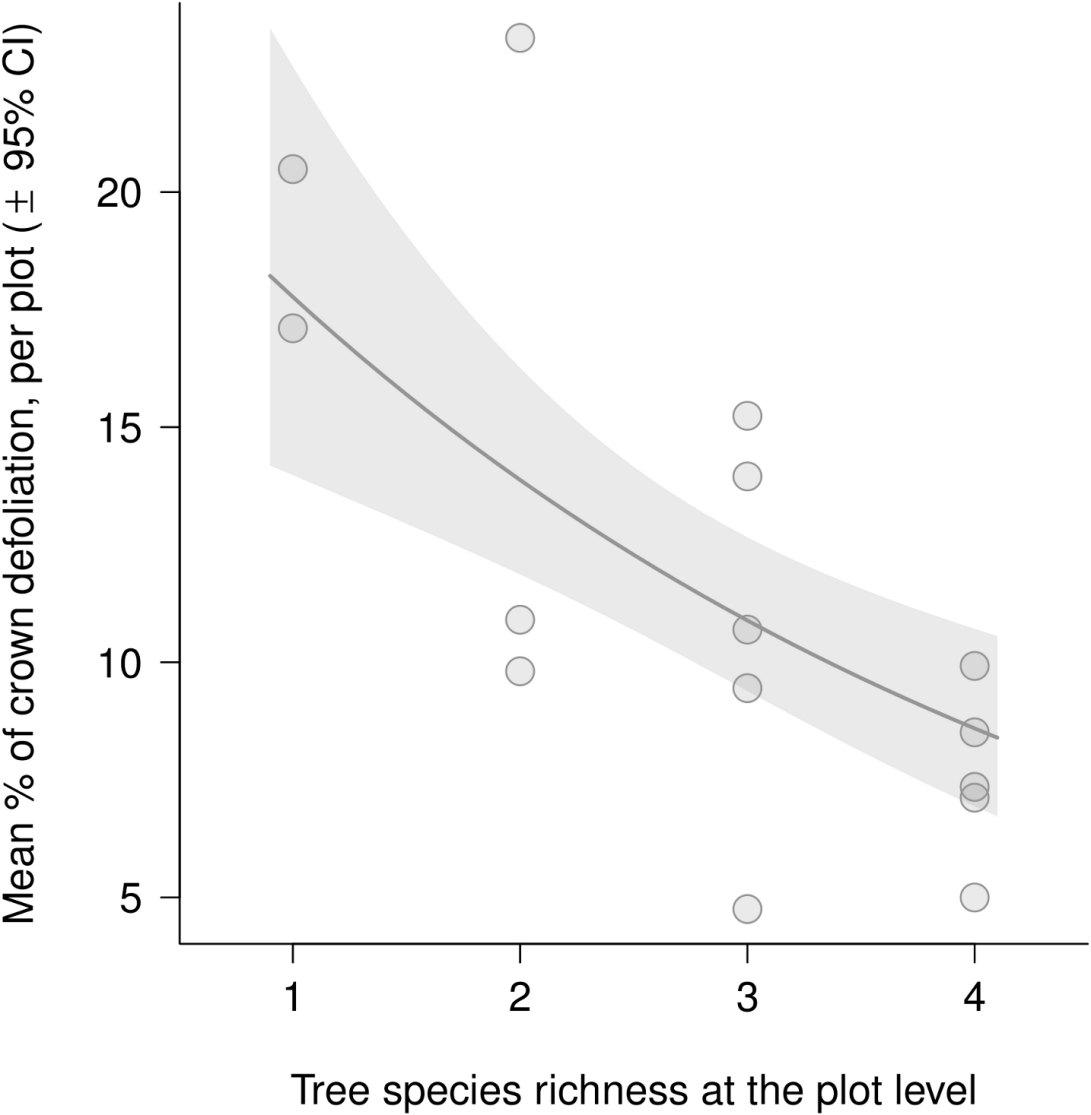
Relationship between total defoliation by *Dryocosmus kuriphilus* and tree species richness at the plot level. Dots represent the mean percentage of total defoliation per plot. The solid line and the shaded area represent predictions from linear mixed models and corresponding confidence interval.

At the plot level, model comparison based on AICc identified tree species richness and Shannon’s diversity index as the variables best fitting defoliation (Table 2). Gall damage was lower with higher tree species richness (*t* = -3.91, *P* = 0.009, Fig 1) and Shannon’s diversity index (*t* = -3.46, *P* = 0.026).

**Table 2:** Results of model selection for the analyses of total defoliation by *Dryocosmus kuriphilus* on chestnut trees in forests with increasing tree species diversity.

LEVEL	MODEL	*K*	AICc	*Δ*_*i*_	*w*_*i*_	Estimate	± SE
**Plot**	***Richness***	*4*	*140*.*38*	*0*.*00*	*0*.*47*	*-0*.*26*	*0*.*07*
(n = 66)	***Shannon’s diversity index***	*4*	*140*.*52*	*0*.*14*	*0*.*44*	*-0*.*59*	*0*.*17*
	**Null**	3	144.61	4.23	0.06		
	**Oak galls**	4	145.66	5.28	0.03		
	**Taxonomic diversity**	4	151.38	11.00	0.00		
	**Proportion of *C*. *sativa***	4	152.76	12.38	0.00		
**Neighbors**	*Tree apparency*	*4*	*71*.*78*	*0*.*00*	*0*.*44*	*0*.*15*	*0*.*04*
(n = 30)	***Shannon’s diversity index***	*4*	*73*.*52*	*1*.*74*	*0*.*18*	*-0*.*70*	*0*.*30*
	**Richness**	4	74.39	2.61	0.12		
	Null	3	74.84	3.06	0.10		
	Shannon’s diversity index	4	74.96	3.18	0.09		
	**Oak galls**	4	77.21	5.43	0.03		
	Richness + Tree apparency	5	77.50	5.72	0.03		
	Richness	4	79.61	7.83	0.01		
	Taxonomic diversity + Tree apparency	5	82.21	10.43	0.00		
	**Taxonomic diversity**	4	83.00	11.23	0.00		
	**Proportion of *C*. *sativa***	4	83.39	11.61	0.00		
	Taxonomic diversity	4	84.31	12.53	0.00		
	Proportion of *C*. *sativa*	4	84.70	12.92	0.00		
	Richness × Tree apparency	6	84.85	13.07	0.00		
	Taxonomic diversity × Tree apparency	6	95.02	23.24	0.00		

All models include plot identity as random factor. Univariate and multivariate models are shown, including their number of estimable parameters (*K*) and their Akaike’s weights (*w*_*i*_). Models within 2 AICc units (*Δ*_*i*_) of the model with the lowest AICc are in *italics*. Estimated parameter values and standard deviations are indicated for these models with *Δ*_*i*_ < 2. Variables in bold are at the plot level and normal typeface variables are at the neighborhood level.

*Null* = Null model; *Richness* = tree species richness; *Shannon’s diversity index* = Shannon index of tree diversity; C. sativa *proportion =* proportion of *Castanea sativa*; *Taxonomic diversity* = Taxonomic diversity index; *Oak galls* = Mean percentage of oak leaves with presence of Cynipid galls; *Tree apparency* = Tree apparency index.

**Fig 2:**
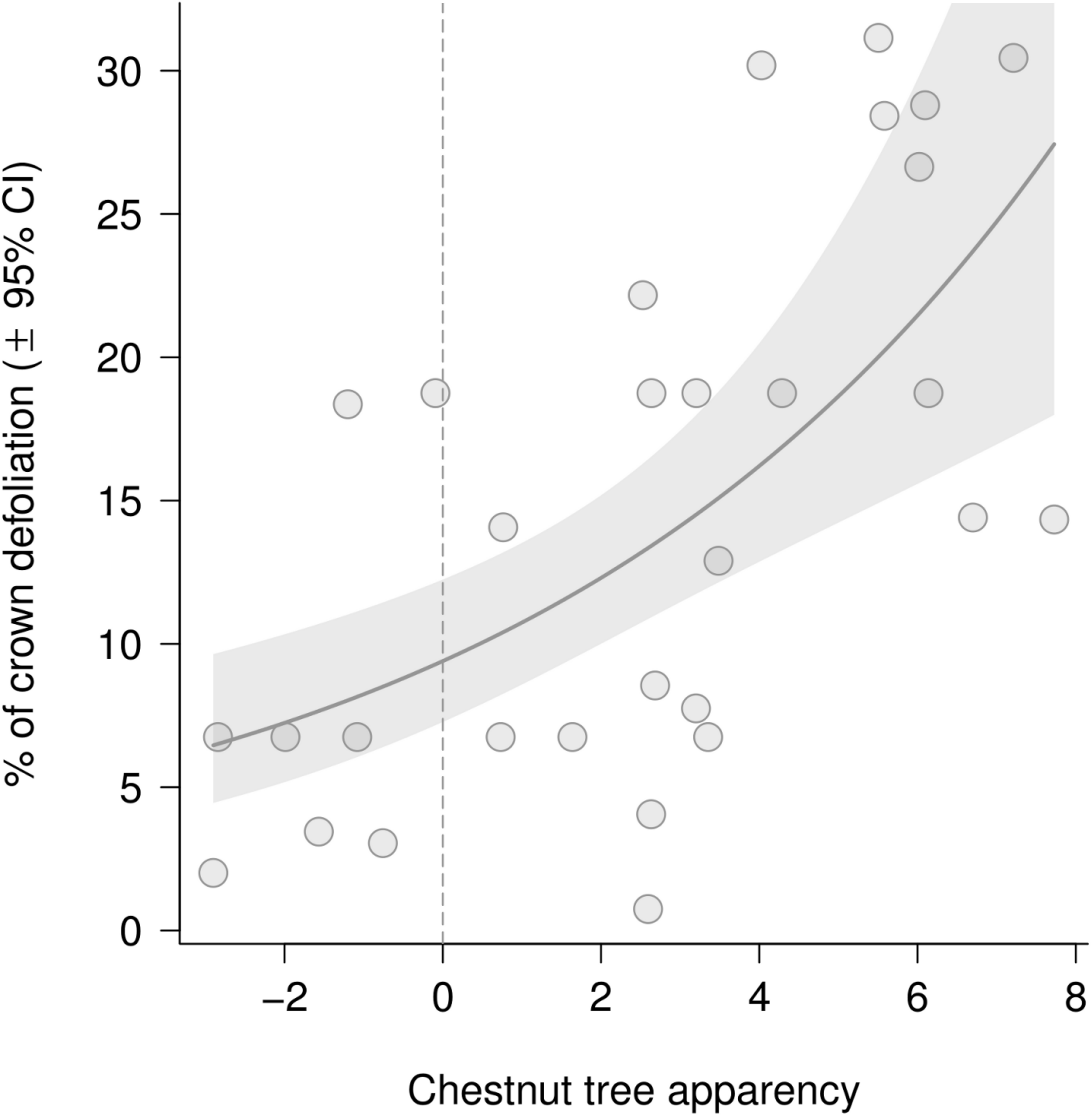
Relationship between total defoliation by *Dryocosmus kuriphilus* and chestnut tree apparency at the neighborhood level. Dots represent the percentage of total tree defoliation. The solid line and shaded area represent predictions by linear mixed models and corresponding confidence intervals. Dots on the left hand side and right hand side represent chestnut trees that were on average shorter and taller than their neighbors, respectively.

At the neighborhood level, none of the multivariate models was retained as best model. Focal chestnut tree apparency at the neighborhood level and Shannon’s diversity index at the plot level were identified as best predictors of total defoliation (Table 2). Damage by *D*. *kuriphilus* was significantly higher on trees with higher apparency: chestnut trees that were taller than their neighbors were subject to twice as much damage as shorter trees on average (*t* = 3.95, *P* < 0.001, Fig 2). Total defoliation was lower with higher Shannon’s diversity index at the plot level, but with a marginal trend toward significance (*t* = -2.36, *P* = 0.062). While tree apparency and Shannon’s diversity index at plot level were correlated (Pearson’s *r* = -0.54, *P* = 0.002), they may have had complementary effects on total defoliation caused by *D*. *kuriphilus*. To test their individual and shared contribution to gall damage, the two variables were included in the same model, fitting tree apparency before, and then after Shannon’s diversity index. Applying sequential decomposition of variance allowed us to test the significance of a second predictor, once the variance explained by the first one was accounted for [57]. Tree apparency had a significant effect whether it was fitted before (*n* = 30, model parameter estimate (± SE) = 0.12 ± 0.04, *P* = 0.011) or after (*n* = 30, 0.12 ± 0.04, *P* = 0.005) the Shannon diversity index. This indicates that, despite the correlation between the two predictors, tree apparency did make an individual contribution to variance in total defoliation. By contrast, Shannon’s diversity index had no significant effect when fitted after tree apparency. The observed effect of tree diversity was therefore mainly due to its correlation with tree apparency.

Observed patterns were robust to the inclusion of outliers as the effects of tree species richness, Shannon’s diversity index and tree apparency were qualitatively the same if outliers were retained in analyses (see Table C in S1 File, see Supporting Information). For analyses at the plot level, tree species richness and Shannon diversity index were still retained in the best models and estimates had exactly the same values (Fig B in S1 File, see Supporting Information). Model rank order was slightly different for analyses at the neighborhood level. Model selection still identified Shannon’s diversity index at the plot level as an important predictor of total defoliation, but univariate model with tree apparency as the sole predictor had a *Δ*_*i*_ > 2. However this discrepancy was due to only one outlier, i.e. one individual tree in a pure chestnut stand which was obviously out of the range of variation in gall damage (Fig C in S1 File, see Supporting Information).Although several explanatory variables were not retained by model comparisons, total defoliation was correlated to some of them. At the plot level, total defoliation was lower with higher percent of oak leaves with Cynipidae galls (*n* = 66, Pearson’s *r* = -0.32, *P* = 0.009), with greater taxonomic diversity (*n* = 66, Pearson’s *r* = -0.38, *P* = 0.002) and with higher proportion of *C*. *sativa* (*n* = 66, Pearson’s *r* = 0.40, *P* = 0.001). At the neighborhood level, total defoliation was lower with higher Shannon diversity index (*n* = 30, Pearson’s *r* = -0.45, *P* = 0.012), and lower proportion of *C*. *sativa* (*n* = 30, Pearson’s *r* = 0.44, *P* = 0.015). All these results consistently suggested that chestnut trees experienced more damage by *D*. *kuriphilus* where grown as pure stands than mixed with other tree species.

## Discussion

Damage caused to chestnut trees by *D*. *kuriphilus* was lower with higher tree species richness or diversity in forest stands. These results demonstrate that tree diversity may contribute to reduce forest invasibility by alien pests, thus confirming associational resistance to the invasive gall wasp. Similar associational resistance to an invasive forest insect was reported for the pine bast scale *Matsucoccus feytaudi* Ducasse at the landscape scale, with a reduction of the spread rate [11] or in stands with only two different tree species [32]. Both invasive forest insects are monophagous, feeding only on tree species in a single genus (*Castanea* and *Pinus* respectively), which is consistent with many studies showing that associational resistance is more likely to occur with specialist herbivores than with generalists [14,16,49].

The “host concentration hypothesis” has been proposed to account for associational resistance to insect herbivores. We tested this hypothesis with *D*. *kuriphilus* by using the proportion of host trees (*C*. *sativa*) in sampled stands as a predictive variable of gall damage. However this explanatory variable was never retained among the best models, whatever the spatial level (plot or neighborhood), suggesting that the dilution of chestnut trees amongst heterospecific trees in mixed forests was not the main driver of associational resistance to this invasive species.

Another mechanism of associational resistance recently gained more attention, the so-called “plant apparency hypothesis” [49]. Host apparency has been defined as the probability of a plant being found by herbivores [61,62]. In mixed forests, the presence of non-host trees can reduce host tree apparency by interfering with the cues that insects use to identify and locate their host. For example, it has been shown that host trees can be hidden by taller heterospecific neighbors, thus decreasing the probability of being visually located by forest insects [49,63,64]. Overall, in our study, chestnut trees that were smaller than their neighbors were less damaged by the invasive gall wasp. However further surveys are needed to confirm that the ability of *D*. *kuriphilus* to locate a host is driven by visual cues. In many phytophagous insects, host recognition is mediated by olfactory cues [65–67] and a mix of host and non-host plant odors may disturb olfactory-guided host choice by specialist herbivores [68–70], as suggested by the “semiochemical diversity hypothesis” [71,72]. Because volatile organic compounds released by host trees and recognized by insect herbivores are likely to diffuse over long distance, disruption of chemical cues by non-host trees could operate at a larger scale than that of visual cues. It is striking that *D*. *kuriphilus* can disperse over long distance and is still capable of finding isolated patches of chestnut trees in invaded landscapes. This could in part explain why the best explanatory model of gall wasp damage at the neighborhood level retained Shannon’s diversity index calculated at the plot level. It is also interesting to note that both tree apparency at the neighborhood level and tree diversity at the plot level significantly reduced the amount of gall damage, suggesting that two complementary associational resistance processes may operate at two nested spatial levels. As demonstrated for other insect herbivores [73], *D*. *kuriphilus* might use olfactory information to identify suitable habitats (i.e. the presence of *Castanea* trees) while using visual cues to locate and colonize a suitable individual host tree.

The enemy release hypothesis [74] predicts that exotic species are successful invaders in the new range because specialist natural enemies were left behind in their native range [75]. However the lack of native enemies might be compensated for by the presence of generalist enemies able to shift onto the new host in the area of introduction [41,76]. Yet several authors reported that predators and parasitoids are more abundant and more diverse in species rich plant communities [27,28,77], thus increasing the chance that these communities contain species able to prey on new alien hosts. This was the case with the maritime pine bast scale *Matsucoccus feytaudi* in its invaded range (Corsica). There, a native predatory bug, *Elatophilus nigricornis* Zetterstedt, which was only present in mixed stands of maritime pine and black pine, was able to shift onto the invasive pest and control its populations [32]. Here, we investigated a similar process by estimating the abundance of cynipid galls on oak trees mixed with chestnut trees. Several studies have already shown that parasitoids native to Italy and emerging from galls on oaks were able to parasitize *D*. *kuriphilus* [42,43,45,46]. In our study, gall damage by *D*. *kuriphilus* was negatively correlated with gall abundance on *Quercus* trees suggesting a potential involvement of native parasitoids emerging from oak galls in chestnut gall wasp control. Currently, the parasitoid *T*. *sinensis*, originating from Japan, is used as classical biological control agent against *D*. *kuriphilus* in Europe. But risks associated with the use of this exotic parasitoid need further investigations to limit negative effects on environment such as hybridization with native parasitoids or spillover onto native gall insects [78]. Promoting native parasitoids through the mixture of chestnuts and oaks (i.e. conservation biological control) could be then a better way to prevent damage caused by the Asian chestnut gall wasp. However, in our study, the abundance of Cynipidae galls on oaks was not retained by model selection suggesting that either natural enemies were not effective biological control agents or that the measure used as a proxy (percentage of oak leaves with galls) misestimated their abundance. There is therefore a need for better sampling both chestnut and oak cynipid galls in order to more accurately estimate their level of parasitism and also identify the parasitoids species really involved in horizontal transfers in mixed stands.

The magnitude of associational resistance to specialist herbivores has been shown to increase with dissimilarity among host and non-host trees, for which phylogenetic distance is commonly used as a proxy [16,79]. In our study, accounting for the identity of tree species associated with chestnut in mixed forests did not provide much additional explanation for the degree of damage caused by the gall wasp, since taxonomic diversity was not retained in model selection. However, the taxonomic diversity index was calculated with only broadleaved species and the three *Quercus* species were at the same taxonomic distance from *C*. *sativa* or *O*. *carpinifolia*. The variation in taxonomic diversity was therefore probably too low to allow the detection of a phylogenetic signal in the diversity-invasibility relationship. It would be more interesting to test this effect after incorporating mixtures of chestnut and conifers (such as *Pinus pinaster* Aiton, present in Tuscany) or other broadleaved species more phylogenetically distant from *C*. *sativa* in the tree diversity gradient.

Recently Liebhold et al. [3] demonstrated that the rate of establishment of invasive pest insects in the US was positively correlated with tree species richness, explaining that it increased the probability of finding a suitable host species. This does is not contradict our finding concerning the lower invasibility of mixed forests. It is simply a further example of the “invasion paradox” [80], which accounts for both negative and positive relationships between native biodiversity and invasions of exotic species. The resolution of this paradox depends on taking the spatial scale into account, as positive associations between native and exotic species richness are observed at large spatial scales (i.e. landscape to continent), and negative associations at fine scales (community scale). We therefore suggest that (i) the successful establishment of an invasive forest pest in a new country may increase with higher γ diversity [81] of trees, as it would increase the probability of finding a suitable new host species, whereas (ii) the rate of development and spread of invasive pest populations may be reduced by a higher α diversity [81] of trees, due to complementary associational resistance mechanisms such as lower host tree availability, accessibility, and higher top-down control by native natural enemies.

## Conclusions

By linking biodiversity and ecosystem functioning concepts with the invasion biology framework, we demonstrated that, at the stand level, tree diversity has the potential to reduce the impact of invasive forest pests. Our results also suggest that more than species richness *per se*, the compositional and structural characteristics of mixed forests are critical to the provision of invasive pest regulation. However, further research is needed to disentangle the ecological mechanisms underlying the diversity-invasibility relationship, e.g. host accessibility or quality *vs* top-down regulation by natural enemies.

## Supporting Information

S1 File Fig A. Schematic representation of a *Castanea sativa* crown damaged by *Dryocosmus kuriphilus* galls. The crown size is arbitrary set equal to 100 cells. Each crown part (in sunlight and in shade, distinguished by the white line) contains dead branches (black cells), defoliated areas (grey cells) and areas with intact leaves (empty cells). […] Table A. Damage caused by Dryocosmus kuriphilus assessed on 70 Castanea sativa trees and explanatory variables calculated at plot and neighborhood levels. […] Table B. *R* syntax for each mixed model used in the model comparison method based on Akaike’s information criterion (Burnham & Anderson 2002). Table C. Results of model selection for the analyses of defoliation by *Dryocosmus kuriphilus* on chestnut trees in forests with increasing tree species diversity using complete data set (i.e. with outliers). Fig B. Relationship between total defoliation by *Dryocosmus kuriphilus* and tree species richness at the plot level using complete data set (i.e. with outliers). Dots represent the mean percentage of total defoliation per plot. The solid line and the shaded area represent predictions from linear mixed models and corresponding confidence interval. Fig C. Relationship between total defoliation by *Dryocosmus kuriphilus* and chestnut tree apparency at the neighborhood level using complete data set (i.e. with outliers). Dots represent the percentage of total tree defoliation. The solid line and shaded area represent predictions by linear mixed models and corresponding confidence intervals. Dots on the left hand side and right hand side represent chestnut trees that were on average shorter and taller than their neighbors, respectively.
(DOCX)
