# Supplementary material for: Tree Diversity Limits the Impact of an Invasive Forest Pest
Source: PLoS One. 2015 Sep 11;10(9):e0136469. doi: 10.1371/journal.pone.0136469 (PMC4567311; doi:10.1371/journal.pone.0136469)
Supplement: S1 File — Dots represent the percentage of total tree defoliation. The solid line and shaded area represent predictions by linear mixed models and corresponding confidence intervals. Dots on the left hand side and right hand side represent chestnut trees that were on average shorter and taller than their neighbors, respectively. (DOCX) [file pone.0136469.s001.docx]

**Tree diversity limits the impact of an invasive forest pest**

Virginie Guyot^1,4*^, Bastien Castagneyrol^3,4^, Aude Vialatte^1,2^, Marc Deconchat^1^, Federico Selvi^5^, Filippo Bussotti^5^, Hervé Jactel^3,4^

^1^ INRA, DYNAFOR, UMR 1201, 31326 Castanet Tolosan, France

^2^ Université de Toulouse, INPT-ENSAT, DYNAFOR, UMR 1201, 31326 Castanet Tolosan, France

^3^ Université de Bordeaux, BIOGECO, UMR 1202, 33405 Talence, France

^4^ INRA, BIOGECO, UMR 1202, 33610 Cestas, France

^5^ Università di Firenze, DISPAA, Laboratori di Botanica, 50144 Florence, Italy

* E-mail: virginie.guyot@ensat.fr

**Fig A. Schematic representation of a *Castanea sativa* crown damaged by *Dryocosmus kuriphilus* galls.** The crown size is arbitrary set equal to 100 cells. Each crown part (in sunlight and in shade, distinguished by the white line) contains dead branches (black cells), defoliated areas (grey cells) and areas with intact leaves (empty cells).

Damage assessment did not include dead branches in the shady part of tree crown. Total percent of defoliation (here, *T_D_* = 28/80 = 0.35) and total proportion of damaged crown (*T_DC_* = 40/100 = 0.40) were calculated based on the following variables:

*T_DBL_*: total proportion of dead branches in the part of the crown exposed to sunlight

*P_CL_*: proportion of the crown part exposed to sunlight (= 60/100, i.e. all cells above the white line)

*P_DBL_*: proportion of dead branches in the crown part exposed to sunlight (= 12/60, i.e. all dark cells above the white line)

*P_DBS_*: proportion of dead branches in the crown part in shade (= 8/40, i.e. all dark cells below the white line)

*P_ACL_*: proportion of the living crown exposed to sunlight (= 48/80, i.e. all empty and grey cells above the white line)

*P_DL_*: proportion of defoliation in the crown part exposed to sunlight (= 16/48, i e. all grey squares above the white line)

*P_DS_*: proportion of defoliation in crown part in shade (= 12/32, i.e. all grey squares below the white line)

And equations are:

$T_{DBL}=P_{CL}\times P_{DBL}=\frac{60}{100}\times\frac{12}{60}=\frac{12}{100}=0.12$ eqn 1

$T_{D}=P_{ACL}\times P_{DL}+\left( 1-P_{ACL} \right)\times P_{DS}=\frac{48}{80}\times\frac{16}{48}+\left( 1-\frac{48}{80} \right)\times\frac{12}{32}=\frac{28}{80}=0.35$ eqn 2

$P_{ACL}=\frac{P_{CL} (1- P_{DBL})}{P_{CL} \left( 1- P_{DBL} \right) + \left( 1- P_{CL} \right) (1- P_{DBS})}=\frac{\frac{60}{100} \left( 1- \frac{12}{60} \right)}{\frac{60}{100} \left( 1- \frac{12}{60} \right) + \left( 1- \frac{60}{100} \right) \left( 1- \frac{8}{40} \right)}=\frac{48}{80}=0.60$ eqn 3

$$T_{DC}=P_{DL}\times P_{CL}\left( 1-P_{DBL} \right)+P_{DS}\left( 1-P_{CL} \right)\left( 1-P_{DBS} \right)+T_{DBL}$$

$=\frac{16}{48}\times\frac{60}{100}\left( 1-\frac{12}{60} \right)+\frac{12}{32}\left( 1-\frac{60}{100} \right)\left( 1-\frac{8}{40} \right)+\frac{12}{100}=\frac{40}{100}=0.40$ eqn 4

**
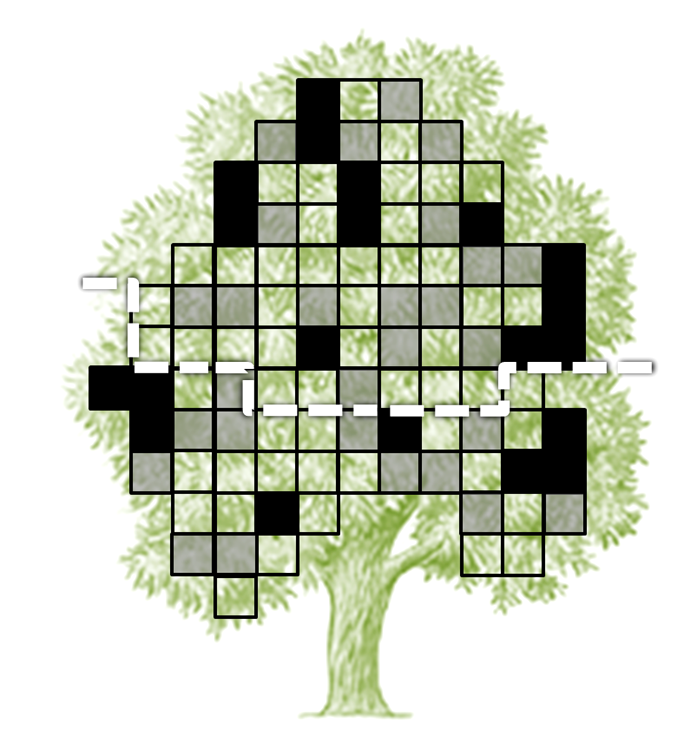
**

**Table A. Damage caused by *Dryocosmus kuriphilus* assessed on 70 *Castanea sativa* trees and explanatory variables calculated at plot and neighborhood levels.**

*T_D_* = Total percent of defoliation

*T_DC_* = Total proportion of damaged crown

*T_DBL_* = Total proportion of dead branches in the part of the crown exposed to sunlight

*"D. kuriphilus"* and "oak galls" are percents of assessed leaves with at least one gall

Shannon diversity and proportion of *C. sativa* are based on relative stem basal area

Taxonomic diversity was calculated using the *taxondive* function in the *ve*gan package in *R* [1]

Tree apparency was based on Castagneyrol et al. [2]

**Table B.** ***R* syntax for each mixed model used in the model comparison method based on Akaike’s information criterion [3].**

| **Level** | **Response variables** | **Fixed factors** | **Random factor** |
| --- | --- | --- | --- |
| **Plot** | CROWN.PLOT ~ | RICHNESS.PLOT | + (1\|ID.PLOT) |
| (n= 70) | CROWN.PLOT ~ | SHANNON.PLOT | + (1\|ID.PLOT) |
|  | CROWN.PLOT ~ | DIST.TAXO.PLOT | + (1\|ID.PLOT) |
|  | CROWN.PLOT ~ | PROP.FOCAL.PLOT | + (1\|ID.PLOT) |
|  | CROWN.PLOT ~ | PC.CYNIPS | + (1\|ID.PLOT) |
|  | CROWN.PLOT ~ | 1 | + (1\|ID.PLOT) |
| **Neighbors** | CROWN.NEIGHB ~ | RICHNESS.PLOT | + (1\|ID.PLOT) |
| (n= 31) | CROWN.NEIGHB ~ | SHANNON.PLOT | + (1\|ID.PLOT) |
|  | CROWN.NEIGHB ~ | DIST.TAXO.PLOT | + (1\|ID.PLOT) |
|  | CROWN.NEIGHB ~ | PROP.FOCAL.PLOT | + (1\|ID.PLOT) |
|  | CROWN.NEIGHB ~ | PC.CYNIPS | + (1\|ID.PLOT) |
|  | CROWN.NEIGHB ~ | RICHNESS.NEIGHB * APPARENCY | + (1\|ID.PLOT) |
|  | CROWN.NEIGHB ~ | RICHNESS.NEIGHB + APPARENCY | + (1\|ID.PLOT) |
|  | CROWN.NEIGHB ~ | RICHNESS.NEIGHB | + (1\|ID.PLOT) |
|  | CROWN.NEIGHB ~ | APPARENCY | + (1\|ID.PLOT) |
|  | CROWN.NEIGHB ~ | SHANNON.NEIGHB | + (1\|ID.PLOT) |
|  | CROWN.NEIGHB ~ | DIST.TAXO.NEIGHB * APPARENCY | + (1\|ID.PLOT) |
|  | CROWN.NEIGHB ~ | DIST.TAXO.NEIGHB + APPARENCY | + (1\|ID.PLOT) |
|  | CROWN.NEIGHB ~ | DIST.TAXO.NEIGHB | + (1\|ID.PLOT) |
|  | CROWN.NEIGHB ~ | PROP.FOCAL.NEIGHB | + (1\|ID.PLOT) |
|  | CROWN.NEIGHB ~ | 1 | + (1\|ID.PLOT) |

*CROWN* = total percent of defoliation (*T_D_*) assessed on crown; *RICHNESS* = tree species richness; *SHANNON* = Shannon index of tree diversity; *PROP.FOCAL* = proportion of *Castanea sativa*; *DIST.TAXO* = Taxonomic diversity index; *PC.CYNIPS* = Mean percentage of oak leaves with presence of cynipid galls; *APPARENCY* = Tree apparency index. Variables with identical names calculated at the plot or neighborhood levels were distinguished by *PLOT* or *NEIGHB* labels. *ID.PLOT* = plot identity.

**Table C. Results of model selection for the analyses of defoliation by *Dryocosmus kuriphilus* on chestnut trees in forests with increasing tree species diversity** using complete data set (i.e. with outliers).

| **LEVEL** | **MODEL** | ***K*** | **AICc** | *Δ_i_* | *w_i_* | **Estimate** | | **± SE** | |
| --- | --- | --- | --- | --- | --- | --- | --- | --- | --- |
| **Plot** | ***Richness*** | *4* | *157.93* | *0.00* | *0.42* | *-0.27* | | *0.08* | |
| (n= 70) | ***Shannon’s diversity index*** | *4* | *158.32* | *0.39* | *0.34* | *-0.59* | | *0.22* | |
|  | **Oak galls** | 4 | 160.25 | 2.32 | 0.13 |  | |  | |
|  | **Null** | 3 | 160.75 | 2.82 | 0.10 |  | |  | |
|  | **Taxonomic diversity** | 4 | 168.22 | 10.30 | 0.00 |  | |  | |
|  | **Proportion of *C. sativa*** | 4 | 169.05 | 11.12 | 0.00 |  | |  | |
| **Neighbors** | ***Shannon’s diversity index*** | *4* | *77.03* | *0.00* | *0.28* | *-0.69* | | *0.36* | |
| (n= 31) | ***Richness*** | *4* | *77.51* | *0.48* | *0.22* | *-0.33* | | *0.14* | |
|  | *Null* | *3* | *77.58* | *0.54* | *0.21* | *2.42* | | *0.19* | |
|  | *Shannon’s diversity index* | *4* | *78.72* | *1.68* | *0.12* | *-0.50* | | *0.42* | |
|  | **Oak galls** | 4 | 79.77 | 2.74 | 0.07 |  | |  | |
|  | Tree apparency | 4 | 79.89 | 2.87 | 0.02 |  | |  | |
|  | Richness | 4 | 82.41 | 5.38 | 0.01 |  | |  | |
|  | Richness + Tree apparency | 5 | 84.99 | 7.96 | 0.00 |  | |  | |
|  | **Taxonomic diversity** | 4 | 86.09 | 9.06 | 0.00 |  | |  | |
|  | Taxonomic diversity | 4 | 86.63 | 9.60 | 0.00 |  | |  | |
|  | **Proportion of C. sativa** | 4 | 86.88 | 9.85 | 0.00 |  | |  | |
|  | Proportion of C. sativa | 4 | 87.63 | 10.60 | 0.00 |  | |  | |
|  | Taxonomic diversity + Tree apparency | 5 | 89.34 | 12.31 | 0.00 |  | |  | |
|  | Richness × Tree apparency | 6 | 91.99 | 14.96 | 0.00 | |  |  |  |
|  | Taxonomic diversity × Tree apparency | 6 | 100.24 | 23.21 | 0.00 |  | |  | |
|  |  |  |  |  |  |  | |  | |

All models include plot identity as random factor. Univariate and multivariate models are shown, including their number of estimable parameters (*K*) and their Akaike’s weights (*w_i_*). Models within 2 AICc units (*Δ_i_*) of the model with the lowest AICc are in *italics*. Estimated parameter values and standard deviations are indicated for these models with *Δ_i_* < 2. Variables in bold are at the plot level and normal typeface variables are at the neighborhood level.

*Null* = Null model; *Richness* = tree species richness; *Shannon’s diversity index* = Shannon index of tree diversity; C. sativa *proportion =* proportion of *Castanea sativa*; *Taxonomic diversity* = Taxonomic diversity index; *Oak galls* = Mean percentage of oak leaves with presence of Cynipid galls; *Tree apparency* = Tree apparency index.

**Fig B. Relationship between total defoliation by *Dryocosmus kuriphilus* and tree species richness at the plot level** using complete data set (i.e. with outliers). Dots represent the mean percentage of total defoliation per plot. The solid line and the shaded area represent predictions from linear mixed models and corresponding confidence interval.


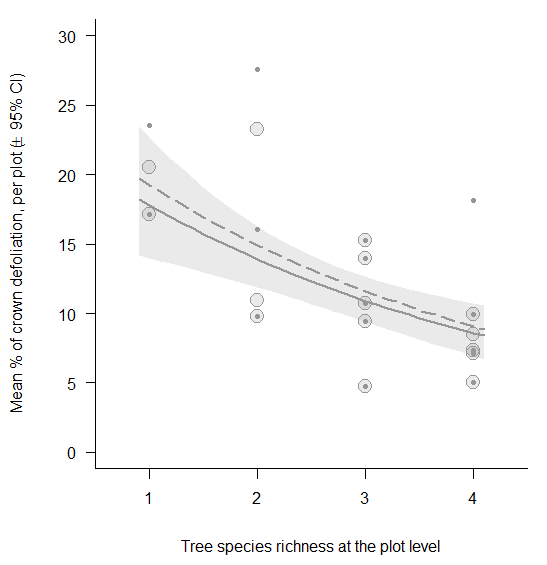


**Fig C. Relationship between total defoliation by *Dryocosmus kuriphilus* and chestnut tree apparency at the neighborhood level** using complete data set (i.e. with outliers). Dots represent the percentage of total tree defoliation. The solid line and shaded area represent predictions by linear mixed models and corresponding confidence intervals. Dots on the left hand side and right hand side represent chestnut trees that were on average shorter and taller than their neighbors, respectively.


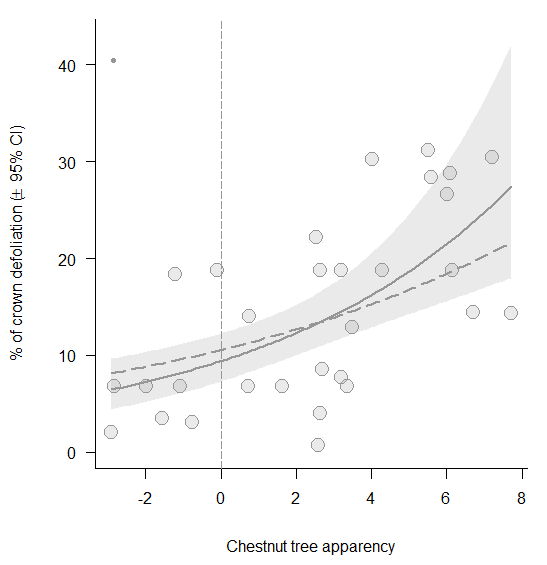


**References**

1. Oksanen J, Blanchet FG, Kindt R, Legendre P, Minchin PR, O’Hara RB, et al. vegan: Community Ecology Package. R package version 2.0-10. 2013.
2. Castagneyrol B, Giffard B, Péré C, Jactel H. Plant apparency, an overlooked driver of associational resistance to insect herbivory. J Ecol. 2013; 101(2):418-429.
3. Burnham KP, Anderson DR. Model selection and multimodel inference: a practical information-theoretic approach. Springer. 2012.
